# Successful kidney transplantation in a patient with late-onset type II Bartter syndrome: A rare case report 

**DOI:** 10.5414/CNCS111808

**Published:** 2025-11-20

**Authors:** Kallappa Baligeri, Mythri Shankar, Dwarak Sampath Kumar, Sreedhara C Gurusiddiah

**Affiliations:** Department of Nephrology, Institute of Nephro-Urology, Bengaluru, India

**Keywords:** Bartter syndrome, kidney transplantation, nephrocalcinosis

## Abstract

Bartter syndrome (BS) is a rare autosomal recessive disorder characterized by inherited salt-losing tubulopathies. Distinguished into six types, each associated with specific genetic mutations, type II is particularly rare in adults and typically presents early. This report documents a rare case of an adult diagnosed with type II Bartter syndrome that progressed to end-stage kidney disease (ESKD) and underwent successful kidney transplantation, marking it a first of its kind in India and only the second globally. The patient, diagnosed in adulthood, experienced a delayed onset of symptoms, including uremia, hypocalcemia, and medullary nephrocalcinosis, which progressed to ESKD. Genetic testing confirmed a homozygous missense mutation in the *KCNJ1* gene. After prolonged hemodialysis, a kidney transplant from a deceased donor resulted in successful graft function and symptom resolution. This case underlines the phenotypic variability of Bartter syndrome and provides critical insights into managing severe, late-onset cases through transplantation.

## Introduction 

Bartter syndrome (BS) is a rare, autosomal recessive disorder with a prevalence of 1 pmp [1]. It includes inherited salt-losing tubulopathies characterized by polyuria, hypokalemia, hypochloremic metabolic alkalosis, and normotensive hyperreninemic hyperaldosteronism. BS is categorized into six types based on the specific gene involved. It is rare for type II to appear during adolescence or adulthood. According to available literature worldwide, there are only seven known instances of late onset type II BS [1, 2] and only one case progressing to end-stage kidney disease (ESKD) [2] (Table 1). In this report, we describe a case of an adult diagnosed with late onset – type II BS progressing to ESKD who further underwent successful kidney transplantation. 

Factors contributing to ESKD in BS include focal segmental glomerulosclerosis, repeated early dehydration, chronic hypokalemia, extensive nonsteroidal anti-inflammatory drug (NSAID) use, nephrocalcinosis, and mutations in the *BSND* gene. Globally, there is only one other documented instance, from Korea [3], of a genetically confirmed BS patient receiving a kidney transplant. To the best of our knowledge, in India this is the first case report of a patient with ESKD due to genetically confirmed diagnosis of BS who has undergone a successful kidney transplantation. 

## Case report 

The male was born to a first-degree consanguineous marriage with a birth weight of 2.6 kg. There were no issues in the perinatal period, including polyhydramnios, and the family history was not significant. 

**Figure 1:**
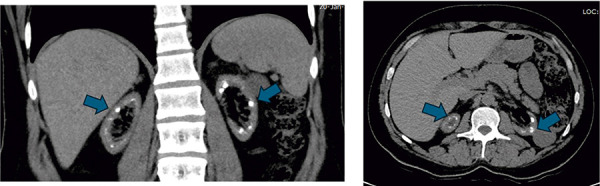
Non-contrast CT abdomen suggestive of bilateral medullary nephrocalcinosis (blue arrows). A: Sagittal section. B: Coronal section. CT abdomen and pelvis: medullary nephrocalcinosis (blue arrows).

The patient, who frequently drank water and urinated during adolescence, was not evaluated until age 32 years when he presented with left ankle pain and was found to have hyperuricemia and kidney dysfunction. Despite being prescribed uricosuric drugs, he was non-compliant. Two years later, he reported generalized weakness, vomiting, and loss of appetite, leading to the initiation of hemodialysis (HD) due to uremic symptoms and was referred to our center. Upon arrival, he was found to be moderately built and nourished (height 172 cm, weight 80 kg) with no obvious skeletal deformities. He was normotensive with normal ECG and 2D echo. Laboratory tests revealed severe uremia (urea: 162 mg%, creatinine: 13 mg%). There was hypocalcemia (8.3 mg%), vitamin D levels were low (25.8 ng/mL) with secondary hyperparathyroidism (321 pg/mL). Other electrolytes were normal. The assessment of the 24-hour urine test was affected due to reduced residual kidney function. The patient’s immunological tests and viral markers returned negative. X-rays of the lumbosacral spine and hands showed no significant findings. CT abdomen and pelvis was suggestive of bilateral medullary nephrocalcinosis (Figure 1). 

He was continued on maintenance HD. Metabolic work-up: 24-hour urine oxalate levels, **s**erum uric acid, and serum cysteine levels were normal. After obtaining informed consent, blood sample was sent for clinical exome sequencing, which revealed a homozygous missense mutation located at exon 2 of the *KCNJ1* gene, chromosome 11, identified as c.658C>T, resulting in the amino acid change p.(Cys220Phe). After 4 years of HD vintage, the patient underwent successful kidney transplantation from a deceased donor of 42 years of age with no comorbidities and terminal creatinine 0.9 mg/dL. The patient had immediate graft function. He was discharged with nadir serum creatinine of 1.0 mg/dL. The post-transplantation period has been uneventful for more than 1 year, with the disappearance of BS. 

## Discussion 

BS exhibits considerable diversity in both its clinical presentation and genetic origins. From a clinical standpoint, BS can be divided into antenatal/neonatal BS and classic BS. Type II BS arises from mutations in *KCNJ1*, which encodes the apical inward-rectifying potassium channel (ROMK). 

A defect in salt reabsorption in the thick ascending limb of loop of henle has two additional consequences that are important in BS, namely (I) a reduction in calcium reabsorption, leading to hypercalciuria and progressive medullary nephrocalcinosis, and (II) a reduction or complete loss of the osmotic gradient in the renal medulla, resulting in isosthenuria. 

To our knowledge, this represents one of the few reported cases of late-onset BS type II and the second only reported case to progress to ESKD (Table 1). Notably, the mutation identified in this patient has also been observed in cases of antenatal BS. The delayed onset in this case points to a remarkable level of phenotypic variability, demonstrating that even identical disease-causing mutations can produce diverse clinical presentations. 

Case reports detailing renal transplantation in patients with BS are scarce [3, 4, 5, 6], most of them are diagnosed clinically as BS (without genetic confirmatory test) as cause of ESKD. In a notable instance [7], preemptive bilateral native nephrectomies followed by renal transplantation were performed before the development of ESKD in a patient with severe, clinically unstable neonatal BS. Across all documented cases, transplantation proved successful, and symptoms of BS were completely resolved post-transplantation, as observed in our patient. 

In summary, BS can be complicated by nephrocalcinosis, which can progress to ESKD. Renal transplantation in patients with BS leads to a complete resolution of BS symptoms. 

## Authors’ contributions 

KB and MS conceptualized and wrote the article. DSK and SCG reviewed that article. 

## Funding 

This case report did not receive any specific grant from funding agencies in the public, commercial, or not-for-profit sectors. 

## Conflict of interest 

The authors declare that there is no conflict of interest regarding the publication of this case report. 

**Table 1.** Cases of late-onset type 2 Bartter syndrome.

Author	Age at presentation	Sex	Clinical presentation	Relevant investigations	Type of mutation in *KCNJ1* gene	DNA sequence change	Amino acid change	Treatment
Huang et al. 2014 [8]	35	Male	Incidental finding of nephrocalcinosis in lumbar spine X-ray done for low back pain	Potassium: 2.8 mEq/L, Creatinine: 1.38 mg/dL, 24-hour calcium excretion: 4.34 mmol/day	Homozygous missense mutation	c.658C>T	p.Leu220Phe	Potassium supplementation and spironolactone
Gollasch et al. 2017 [9]	43	Female	Incidental finding of nephrocalcinosis in ultrasound done during pregnancy	Potassium: 2.8 mEq/L Creatinine: 1.1 mg/dL, 24-hour calcium excretion: 7.5 mmol/day	Compound heterozygous missense mutation	c.197T>A (novel mutation)c.875G>A	p.Ile66Asnp.Arg292Gln	Potassium supplement and angiotensin-converting enzyme inhibitors (ramipril)
Li et al. 2019 [10]	34	Female	Weakness, persistent polyuria and polydipsia; weight and height normal	Potassium: 2.4 mEq/L Creatinine: 1.1 mg/dL, 24-hour calcium excretion: 6.9 mmol/day	Compound heterozygous missense mutation	c.701C>T (novel mutation)c.212C>T	p.The234Ilep.Thr71Met	Potassium supplementation
Sharma et al. 2011 [11]	8.5	Female	Persistent polyuria and polydipsia; fifth percentile for weight and height	Potassium: 2.5 mEq/ L, Creatinine: 0.5 mg/dL Ca/Creatinine: 0.91 mg/mg (normal < 0.2)	Novel compound heterozygous mutation	c.268G>Tc.632T>G	p.Arg90Trpp.Ile211Ser	Potassium supplementation and nonsteroidal anti-inflammatory drugs
Elfert et al. 2020 [12]	26	Male	Weakness, persistent polyuria and polydipsia; weight and height normal	Potassium: 1.7 mEq/L, Creatinine: 1.08 mg/dL, 24-hour calcium excretion: 3.5 mmol/day	Homozygous missense mutation	c.658C>T	p.Leu220Phe	Potassium supplement and aldosterone antagonists
Gagger et al. [2] 2023	34	Male	Nausea, vomiting, and decreased appetite, polydipsia, polyuria, nephrocalcinosis	Urea: 139 mg%, creatinine: 10 mg% 3.28 meq/L), hypocalcemia (5.0 mg%), and hyperphosphatemia (7.0 mg%)	Homozygous missense mutation	c.500 G>A	p.Gly167Glu	End-stage kidney disease – initiated on maintenance hemodialysis
Tian et al. 2022 [1]	34	Female	Polydipsia, polyuria, generalized body numbness, nephrocalcinosis	Creatinine: 0.7 mg/dL serum potassium level of 2.9 mmol/L, accompanied by hypomagnesemia and hypocalcemia	compound heterozygous missense mutation	c.619C>A c.922C>T	p. Leu207Ile p. Cys308Arg	Potassium supplement and aldosterone antagonists
Present case	34	Male	Nausea, vomiting, fatigue, weight and height normal. Bilateral medullary nephrocalcinosis	Creatinine: 13 mg/dL	Homozygous missense mutation	c.658C>T	p.(Cys220Phe)	End-stage kidney disease – initiated on maintenance hemodialysis
